# Integrated management of dry eye–depression comorbidity: mechanistic insights and temporal therapeutic strategies

**DOI:** 10.3389/fmed.2026.1829273

**Published:** 2026-06-02

**Authors:** Te-du Yang, Chuan-long Wu, Xiao-juan Li, Shao-rong Hu, Jing Zang

**Affiliations:** 1 Department of Comprehensive Ophthalmology, Shenzhen Shendong Aier Eye Hospital, Shenzhen, China; 2 Department of Special Examinations, Shenzhen Shendong Aier Eye Hospital, Shenzhen, China

**Keywords:** comorbidity, depressive disorder, dry eye disease, eye-brain axis, integrated care, patient-centered care, phenotypic stratification, temporal sequencing

## Abstract

The frequent co-occurrence of dry eye disease (DED) and depressive disorder imposes a significant dual burden on patient quality of life and healthcare systems. Current fragmented, discipline-specific care models remain inadequate for addressing this clinical reality. The core challenge lies in the absence of integrated management frameworks for comorbid presentations, with particular neglect of treatment sequencing that often compromises therapeutic outcomes. This perspective article proposes that effective management of DED-depression comorbidity should build upon shared pathophysiological mechanisms—including neurogenic inflammation and eye-brain axis dysfunction—to construct an integrated mind–body therapeutic paradigm. We present a comprehensive framework encompassing stratified phenotypic assessment, a stepwise therapeutic ladder, and a dynamic temporal optimization strategy guided by bidirectional biological and psychological interactions. This integrated paradigm holds promise for transcending disciplinary boundaries, enabling precision treatment selection, and ultimately improving long-term patient outcomes through holistic, patient-centered care.

## 1 Introduction

### 1.1 Dual burden of clinical reality

The frequent co-occurrence of dry eye disease (DED) and depressive disorder presents a growing clinical challenge. Epidemiological studies consistently demonstrate that depressive symptoms are significantly more prevalent among DED patients than in the general population, with meta-analyses reporting odds ratios between 1.5 and 2.5 (1, 2). This association is bidirectional; individuals with depression also face increased risk of developing DED (3). Rather than viewing these conditions as merely coincident, we propose that DED and depression share fundamental biological pathways-including neurogenic inflammation and hypothalamic–pituitary–adrenal axis dysregulation-as well as psychological mechanisms involving symptom perception and illness amplification (4). Recognizing this relationship as a genuine comorbid syndrome with reciprocal influences is essential for advancing both research and clinical practice.

### 1.2 Limitations of current therapeutic approaches

The prevailing model of fragmented, discipline-specific care creates substantial therapeutic challenges. Ophthalmologists appropriately assess objective signs of ocular surface disease but typically omit standardized evaluations of psychological distress that profoundly modulate symptom experience (1). Conversely, mental health professionals address core affective disturbances while often overlooking associated somatic complaints, such as persistent ocular discomfort, that may perpetuate depressive states (5). This disciplinary compartmentalization inevitably compromises treatment outcomes. Critical examination of current clinical guidelines reveals a notable deficiency: they are predominantly developed as single-disease protocols addressing either DED or depression in isolation (6). This absence of integrated guidance leaves clinicians without evidence-based frameworks for managing the complex bidirectional interactions between ocular pathology and mood dysregulation (6).

### 1.3 Purpose and perspective of this article

This perspective article aims to transcend traditional disciplinary boundaries by examining DED-depression comorbidity through an integrated, systems-oriented lens. Rather than merely cataloging established associations, we propose a conceptual framework emphasizing temporal dynamics as critical determinants of therapeutic success. Specifically, we argue that understanding “when to intervene” in the bidirectional trajectory of these conditions may prove as clinically important as determining “what intervention to apply.” By delineating pathophysiological pathways linking ocular surface pathology with mood dysregulation and proposing sequential management strategies (4, 7), this perspective article provides clinicians with practical guidance for navigating the current therapeutic vacuum. We ultimately advocate for a paradigm shift toward integrated approaches that address the whole patient rather than disconnected organ systems.

## 2 Shared mechanisms: bidirectional pathway between dry eye and depression

### 2.1 Shared biological substrates

**Figure 1:**
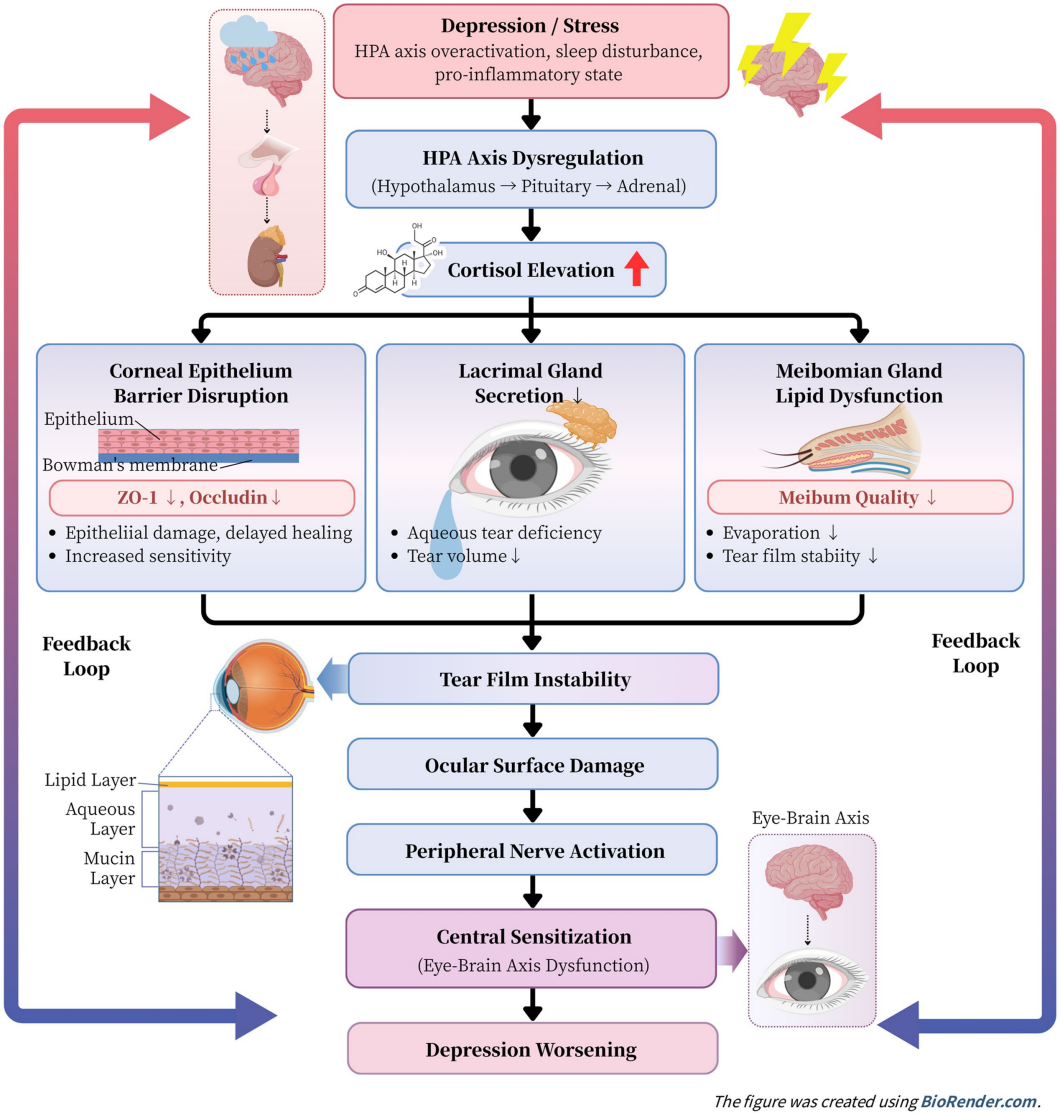
Bidirectional mechanisms linking dry eye disease and depression. HPA axis activation elevates cortisol, disrupting corneal epithelial barriers (e.g., ZO-1, occludin) and impairing lacrimal and meibomian gland function, leading to tear film instability. Peripheral nerve activation and central sensitization reinforce depressive symptoms, forming a pathological feedback loop. Glucocorticoid-mediated ocular surface dysfunction serves as a key mechanistic bridge. Created with BioRender.com.

The frequent co-occurrence of DED and depressive disorder may be partially explained by overlapping biological pathways, particularly systemic inflammation. Persistent low-grade inflammation, characterized by elevated pro-inflammatory cytokines such as interleukin-6 and tumor necrosis factor-alpha, represents a transdiagnostic mechanism underlying both conditions (8). In DED, these mediators drive ocular surface inflammation and tear film disruption; in depression, they contribute to neuroinflammatory processes affecting mood-regulating brain regions (9). Anatomical connections between peripheral and central nervous systems provide another mechanistic link (10). The densely innervated cornea transmits nociceptive signals via the trigeminal pathway to central structures involved in emotional processing (11). Chronic ocular discomfort may therefore sensitize these circuits, perpetuating emotional dysregulation and heightened pain perception. Pharmacological and neuroendocrine factors further modulate this bidirectional relationship. Antidepressant medications, particularly selective serotonin reuptake inhibitors, can adversely affect tear secretion and meibomian gland function (12). Concurrently, hypothalamic–pituitary–adrenal axis dysregulation in depression elevates cortisol levels, compromising tear film stability and corneal epithelial integrity (13). At the molecular level, elevated cortisol may disrupt corneal epithelial barrier integrity by affecting tight junction stability, suppress lacrimal gland secretion through neuroendocrine inhibition, and alter lipid synthesis and secretion within the meibomian glands (14, 15). These combined effects impair tear film homeostasis and promote instability, thereby providing a mechanistic bridge between neuroendocrine stress responses and ocular surface dysfunction (15, 16). These interconnected mechanisms suggest that DED and depression reinforce each other through shared physiological pathways rather than existing as etiologically independent conditions (Figure 1).

### 2.2 Psychological and behavioral vicious cycles

The interaction between DED and depression extends into psychological and behavioral domains, where symptoms of each condition amplify the other through reciprocal feedback mechanisms (5, 17). Ocular discomfort, pain, and foreign body sensation frequently disrupt sleep quality and impair daily functioning, leading to fatigue, reduced social participation, and diminished quality of life—factors well-established as contributors to depressive onset (18, 19). Conversely, depression-associated psychomotor retardation and reduced physical activity decrease blink frequency and meibomian gland function, directly compromising tear film stability and ocular surface health (5). This bidirectional amplification creates a self-perpetuating cycle that complicates clinical management. Cognitive factors further modulate this relationship. Depressed individuals typically exhibit negative cognitive biases, including catastrophizing and selective attention to threatening information (20). When applied to DED symptoms, such patterns lead to exaggerated interpretations of benign ocular sensations as signs of serious disease or irreversible damage (21). These maladaptive appraisals reduce treatment adherence, increase healthcare-seeking behavior without corresponding clinical findings, and diminish satisfaction with otherwise appropriate interventions (22). Understanding these psychological mediators is therefore essential for developing comprehensive management strategies addressing both sensory and emotional dimensions of this comorbid condition.

## 3 Diagnostic reformulation: from symptom recognition to comorbidity stratification

### 3.1 Screening: establishing an integrated perspective in specialty clinics

Effective management of dry eye-depression comorbidity begins with systematic identification in routine practice. In ophthalmology settings, we advocate implementing ultra-brief depression screening tools, such as the Patient Health Questionnaire-2 or Patient Health Questionnaire-9 (PHQ-9), during standard dry eye evaluations (23, 24). These instruments demonstrate acceptable sensitivity and specificity in medically ill populations and require minimal time, making them feasible for busy clinical environments (24). Unrecognized depression in dry eye patients predicts poorer treatment adherence, reduced satisfaction with ocular interventions, and worse functional outcomes (25). Conversely, in psychiatric settings, clinicians should routinely inquire about ocular symptoms, particularly for patients taking medications with anticholinergic effects or those affecting tear secretion (26). Selective serotonin reuptake inhibitors, serotonin-norepinephrine reuptake inhibitors, and tricyclic antidepressants may all influence tear film dynamics (27). Simple questions about ocular discomfort, visual fluctuation, or prior dry eye diagnoses can effectively identify individuals needing ophthalmological referral. This bidirectional screening approach ensures that neither condition remains unrecognized when patients present to single-discipline specialists.

### 3.2 Stratification: toward a mechanism-driven phenotypic classification

Traditional classification systems for DED—distinguishing aqueous-deficient from evaporative subtypes—and for depressive disorders—differentiating unipolar from bipolar forms—provide insufficient guidance for managing comorbid presentations (28, 29). While valuable within their respective specialties, these parallel taxonomies fail to capture the mechanistic heterogeneity underlying the dry eye-depression interface. We therefore propose a provisional integrative phenotyping framework based on dominant pathophysiological mechanisms.

The inflammation-dominant phenotype features prominent ocular surface inflammatory signs alongside depressive presentations with pronounced fatigue, psychomotor retardation, and somatic symptoms (30). Systemic inflammatory markers may be elevated in this subgroup, suggesting shared inflammatory pathophysiology (31).

The neuropathic pain-dominant phenotype presents with significant corneal pain or heightened sensitivity often disproportionate to objective ocular findings (32). These patients demonstrate high anxiety comorbidity rates and may exhibit central sensitization features (1). Central sensitization represents a core mechanistic driver in this phenotype, characterized by amplified nociceptive signaling within the central nervous system (33, 34). This phenomenon contributes to dysfunction of the “eye–brain axis,” whereby peripheral ocular inputs are disproportionately interpreted as pain, reflecting maladaptive central processing rather than purely peripheral pathology (33, 35). From a therapeutic perspective, neuromodulating agents—such as gabapentin—may mitigate symptoms by reducing neuronal hyperexcitability and restoring central pain modulation (36, 37). Standard tear-based therapies are often insufficient in this subgroup, highlighting the need for mechanism-targeted interventions (35, 37).

The iatrogenic or medication-induced phenotype describes patients developing dry eye symptoms following psychotropic medication initiation, particularly agents with anticholinergic properties (38, 39). Temporal relationships between drug initiation and symptom onset provide diagnostic clues.

The behavior or stress-related phenotype encompasses patients whose comorbid presentation emerges during psychological stress accompanied by sleep disturbance and anxiety (40). Behavioral factors, including reduced blink frequency during computer use, may bridge psychological and ocular domains (41).

To further enhance clinical applicability, provisional quantitative criteria may be incorporated into this framework to support phenotype assignment. For instance, an Ocular Surface Disease Index score ≥23—indicative of moderate-to-severe dry eye—combined with a PHQ-9 score in the mild-to-moderate range (5–14) may support classification into the inflammation-dominant phenotype when objective inflammatory signs are present (1, 42). In contrast, individuals with similarly elevated symptom scores but minimal observable ocular findings may be more appropriately categorized as neuropathic pain-dominant, reflecting symptom–sign dissociation (35). The medication-induced phenotype should be identified primarily through a clear temporal association with antidepressant initiation, whereas the stress-related phenotype may be inferred from concurrent elevations in PHQ-9 scores alongside sleep disruption and identifiable behavioral or psychosocial triggers (4). These criteria are intended as pragmatic clinical guides rather than definitive diagnostic thresholds and require validation in future prospective studies.

This provisional framework requires empirical validation but offers clinical utility by directing attention toward dominant mechanisms that may guide personalized treatment selection for patients with dry eye-depression comorbidity (Table 1).

**Table 1:** Phenotype-guided clinical management of dry eye–depression comorbidity.

Phenotype	Key clinical features	Dominant mechanism	Suggested diagnostic indicators	Preferred entry point	Targeted interventions
Inflammation- dominant	Ocular surface inflammation, dryness, fatigue, somatic depressive symptoms	Systemic and ocular surface inflammation	OSDI ≥ 23 + PHQ-9: 5–14 with objective inflammatory signs (e.g., TBUT↓, corneal staining)	Ophthalmology-led	Topical anti-inflammatory therapy (e.g., cyclosporine), omega-3 supplementation, tear substitutes
Neuropathic pain-dominant	Severe ocular pain or burning with minimal clinical signs; high symptom–sign mismatch	Central sensitization, eye–brain axis dysregulation	OSDI ≥ 23 with minimal objective signs; disproportionate pain ± anxiety	Pain/multidisciplinary care	Neuromodulators (e.g., gabapentin), CBT, pain-focused management
Medication- induced (iatrogenic)	Onset of dry eye symptoms after antidepressant use	Drug-induced tear dysfunction (anticholinergic effects)	Clear temporal association with antidepressant initiation	Psychiatry-led (with ophthalmology collaboration)	Medication adjustment (under supervision), tear substitutes, secretagogues
Stress- behavior related	Sleep disturbance, stress, anxiety, digital overuse, fluctuating symptoms	Behavioral dysregulation, neuroendocrine stress response	Elevated PHQ-9 + sleep disturbance+behavioral triggers (e.g., screen exposure)	Integrated care	Sleep optimization (CBT-I, melatonin), lifestyle modification, blink training

OSDI, ocular surface disease index; PHQ-9, patient health questionnaire-9; TBUT, tear break-up time; CBT, cognitive behavioral therapy; CBT-I, cognitive behavioral therapy for insomnia.

## 4 Pathway design: constructing an integrated mind-eye therapeutic ladder

### 4.1 Foundational layer: collaborative interventions for shared problems

**Figure 2:**
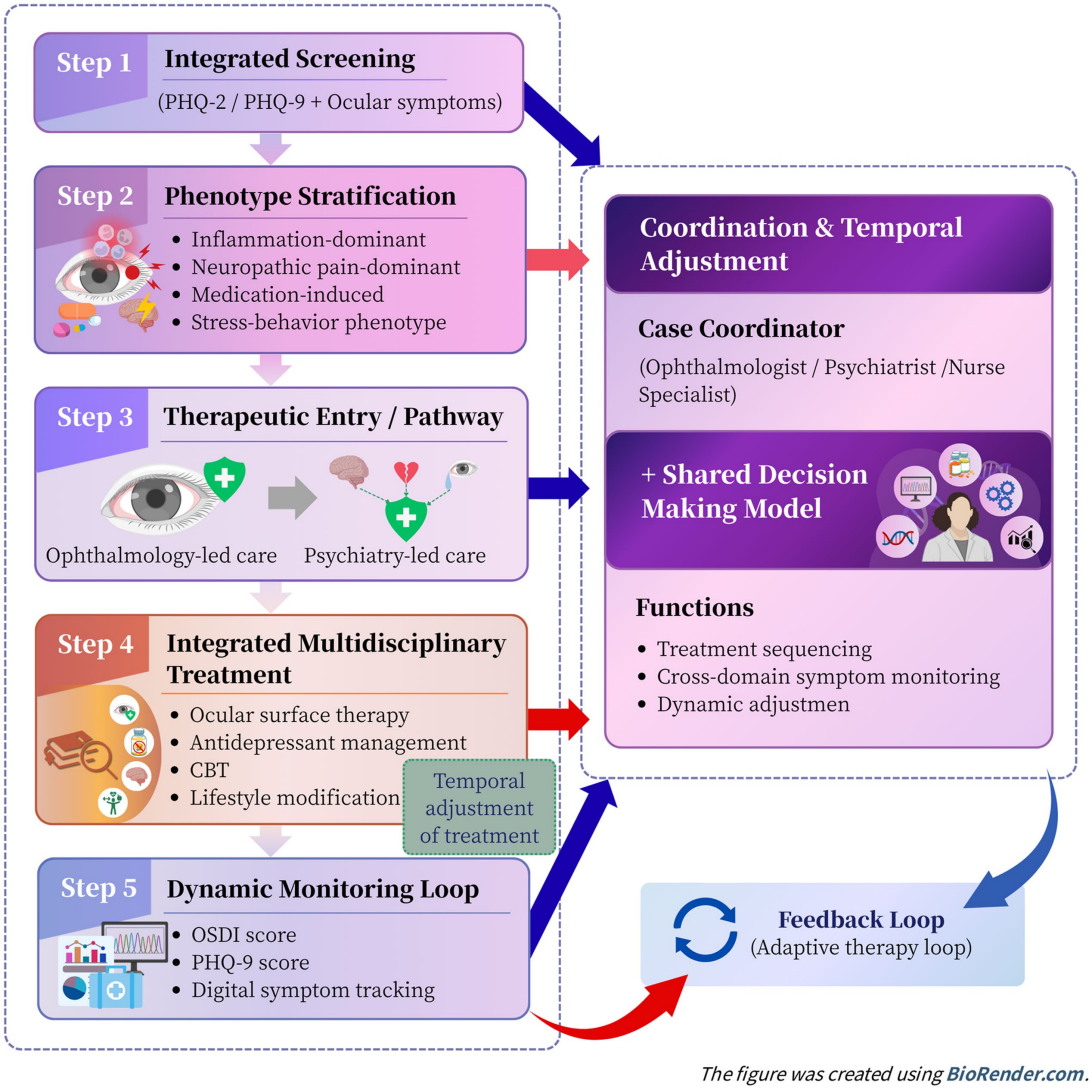
Integrated stepwise management pathway for dry eye-depression comorbidity. A structured clinical pathway involving screening, phenotyping, and multidisciplinary intervention. A designated case coordinator (e.g., ophthalmologist, psychiatrist, or nurse specialist) manages treatment sequencing, cross-domain monitoring, and provider communication. Shared decision-making ensures patient-centered, adaptable care, with a feedback loop for continuous reassessment and optimization. Created with BioRender.com.

Integrated care for dry eye-depression comorbidity follows a structured, stepwise clinical pathway that integrates screening, phenotypic stratification, and multidisciplinary intervention (Figure 2). Comprehensive patient education serves as an essential first step. Clinicians should clearly communicate the bidirectional relationship between ocular discomfort and emotional distress, enabling patients to conceptualize their symptoms as interconnected manifestations of a shared pathophysiological process rather than isolated conditions (43). This cognitive reframing may strengthen therapeutic alliance and enhance adherence to subsequent interventions.

Lifestyle modification represents a foundational component of this integrated approach. Among these, sleep regulation warrants particular emphasis due to its central role in both ocular surface homeostasis and mood stability (44). Disturbances in sleep are closely associated with increased inflammatory activity and impaired emotional regulation, thereby contributing to symptom persistence across both domains.

Beyond general sleep hygiene measures, more targeted interventions may be considered to optimize therapeutic outcomes. These include cognitive behavioral therapy for insomnia, which addresses maladaptive sleep-related cognitions and behaviors, as well as short-term melatonin supplementation to support circadian rhythm stabilization (45, 46). Importantly, insufficient or disrupted sleep has been shown to elevate pro-inflammatory cytokine levels, which may exacerbate ocular surface inflammation and tear film instability (47–49). Through this inflammatory pathway, sleep dysregulation may preferentially contribute to the inflammation-dominant phenotype, thereby providing a mechanistic rationale for prioritizing sleep-focused interventions in this subgroup (47, 50).

Regular aerobic exercise provides additional synergistic benefits by promoting brain-derived neurotrophic factor secretion—supporting mood regulation—while also enhancing meibomian gland function through improved microcirculation (51). Nutritional strategies further complement this approach. Diets enriched in omega-3 fatty acids and reduced in pro-inflammatory components may help attenuate systemic inflammation, thereby benefiting both ocular and psychological symptoms (52).

Collectively, these foundational interventions require minimal specialization, can be implemented across clinical settings, and serve as a critical entry point for interrupting shared pathological mechanisms before progressing to more targeted therapies.

### 4.2 Specialty layer: entry point selection based on stratification

Following initial assessment, the appropriate entry point into the therapeutic ladder should be determined by dominant clinical features and their relative severity. For patients presenting with prominent dry eye symptoms accompanied by mild to moderate depressive features, ophthalmology-led care with psychiatric collaboration represents the appropriate pathway (4). In this model, ophthalmologists optimize anti-inflammatory treatment while incorporating brief behavioral interventions, such as combining eyelid hygiene with mindfulness-based practices. Regular mood monitoring ensures early identification of deterioration requiring stepped care (53). Conversely, for patients with severe depression—particularly those with suicidal ideation—or those whose dry eye symptoms appear primarily medication-induced, psychiatry-led care with ophthalmological collaboration is indicated (54). In this pathway, psychiatrists prioritize mood stabilization, consider medication adjustments to minimize ocular side effects, and coordinate with ophthalmologists for targeted symptom management. This flexible approach respects each specialty’s expertise while ensuring neither condition is neglected.

### 4.3 Integrated layer: multidisciplinary collaboration for complex comorbidity

Patients with complex or treatment-refractory comorbidity require genuine multidisciplinary collaboration beyond parallel care (55). Physical treatments for dry eye may offer unanticipated psychological benefits through shared neural pathways (6). Intense pulsed light therapy, for instance, may modulate local nerve function in ways that indirectly influence central pain processing and emotional regulation, though this requires further investigation (4). Psychological interventions similarly warrant adaptation. Cognitive behavioral therapy protocols should address the specific “discomfort-catastrophizing-avoidance” cycle characteristic of this comorbidity, helping patients distinguish catastrophic interpretations from realistic appraisals of ocular sensations (56). Pharmacological management demands careful collaboration. When dry eye symptoms significantly impair quality of life, psychiatrists should consider, in consultation with ophthalmologists, whether alternative antidepressants with more favorable ocular profiles might be appropriate, or whether adjunctive secretagogues could mitigate medication-induced dryness without compromising psychiatric stability (25). In practical implementation, an organized coordination structure is essential to translate multidisciplinary principles into clinical practice (57). A designated case coordinator—such as a primary ophthalmologist, psychiatrist, or a trained nurse specialist—may oversee temporal treatment adjustments, monitor cross-domain symptom trajectories, and facilitate communication among providers (58). This coordinated model can be further strengthened through shared decision-making frameworks, ensuring that therapeutic strategies remain patient-centered while maintaining consistency across disciplines (59). Collectively, this integrated framework underscores that optimal management of DED–depression comorbidity depends not only on combining therapeutic modalities, but also on structuring care delivery through coordinated, patient-centered, and dynamically adaptable systems.

## 5 Temporal optimization: a dynamic navigation system for integrated pathways

### 5.1 Establishing priority principles for temporal sequencing

Effective management of dry eye-depression comorbidity requires determining not only which interventions to use but also their optimal sequence. We propose three priority principles to guide temporal decision-making.

First, safety takes precedence. When patients present with severe depression, particularly suicidal ideation or self-harm risk, psychiatric intervention should begin immediately, regardless of ocular symptom severity (60). Importantly, any modification to antidepressant therapy must be conducted under formal psychiatric supervision (61, 62). Unsupervised discontinuation, dose reduction, or medication switching may precipitate withdrawal syndromes or depressive relapse, thereby introducing risks that may outweigh potential improvements in ocular surface symptoms (62, 63). This consideration underscores that treatment sequencing must not compromise psychiatric stability while addressing ocular comorbidity (61, 62).

Second, etiological attribution should guide sequencing. When dry eye symptoms clearly result from medications with known ocular effects—such as isotretinoin or antidepressants with anticholinergic properties—modifying the causative agent should precede or accompany symptomatic treatment (64). In such contexts, therapeutic decision-making should follow a collaborative model involving both psychiatrists and ophthalmologists to balance psychiatric efficacy with ocular tolerability. Adding artificial tears without addressing the underlying pharmacological cause is often insufficient (65).

Third, symptom severity and functional impact should inform timing. When severe ocular pain disrupts sleep or precipitates emotional crises, aggressive ocular surface therapy may need to proceed concurrently with or before antidepressant initiation (44). Uncontrolled physical discomfort can undermine psychiatric treatment adherence and efficacy (2).

### 5.2 Constructing a dynamic monitoring-feedback-adjustment loop

Temporal optimization requires continuous assessment and responsive adjustment rather than static treatment plans. We introduce the concept of the “therapeutic window of vulnerability,” referring to periods when patients face elevated risk of treatment discontinuation or symptom exacerbation. The initial 2 to 4 weeks following antidepressant initiation represent a critical window (66). During this period, therapeutic benefits have not yet emerged, while ocular side effects may become apparent. Patients experiencing increased ocular discomfort during this window may discontinue psychiatric treatment prematurely (67). Proactive ophthalmological support during this vulnerable period—through intensified lubrication or anti-inflammatory therapy—may prevent treatment dropout (68). Dynamic assessment tools facilitate this adaptive approach. Brief symptom diaries or digital applications enabling simultaneous tracking of ocular surface symptoms using the Ocular Surface Disease Index and mood ratings via the Patient Health Questionnaire-9 allow clinicians to monitor both domains longitudinally and make individualized adjustments (69, 70).

### 5.3 Temporal strategies for addressing clinical heterogeneity: illustrative scenarios

The application of temporal principles varies according to clinical phenotype. For the neuropathic pain-dominant phenotype, characterized by severe corneal pain often disproportionate to objective signs, pain management assumes temporal priority (71). Initiating neuromodulating agents, such as gabapentin or low-dose tricyclic antidepressants, may simultaneously address ocular discomfort and provide mood stabilization (72).

For the inflammation-dominant phenotype, therapeutic sequencing should be guided by the interaction between ocular surface inflammation and systemic symptom burden (10, 73). Although direct longitudinal trials comparing different treatment sequencing strategies remain limited, converging evidence indicates that ocular surface inflammation is closely associated with fatigue and somatic symptomatology, both of which are key contributors to depressive severity (74, 75). In this context, prioritizing anti-inflammatory therapy at the ocular level may reduce peripheral inflammatory signaling and, indirectly, mitigate symptom clusters that overlap with depressive presentations. Accordingly, anti-inflammatory interventions for DED may be reasonably positioned as an initial therapeutic step in patients with a predominant inflammatory profile (28, 76). This sequencing approach should be interpreted as hypothesis-driven rather than definitive, and its clinical superiority over concurrent or reversed strategies requires validation in future prospective and longitudinal studies.

For the medication-induced phenotype, the sequence begins with psychiatric medication review (38). Adjusting antidepressant selection toward agents with more favorable ocular profiles, while maintaining psychiatric stability, represents the foundational step before adding targeted dry eye therapies (77).

Collectively, these illustrative scenarios underscore that phenotypic-guided temporal sequencing is not merely a matter of treatment order, but a mechanism-informed strategy aimed at interrupting dominant pathological loops. By aligning intervention timing with underlying biological and behavioral drivers, this approach may enhance therapeutic synergy and improve overall clinical outcomes in dry eye–depression comorbidity.

## 6 Challenges and future directions

### 6.1 Practical barriers

Despite the theoretical promise of integrated management for dry eye-depression comorbidity, several practical barriers hinder its clinical implementation (1, 2). Healthcare system structures present significant challenges. Effective multidisciplinary collaboration requires coordinated referral pathways, shared visit scheduling, and integrated billing mechanisms—infrastructure that remains underdeveloped in most practice settings (78). As a result, patients frequently navigate fragmented care pathways across independent specialty clinics, often without structured communication between providers, leading to discontinuity in treatment and suboptimal outcomes (2).

In addition to structural limitations, knowledge gaps across disciplines further impede integration. Ophthalmologists may have limited familiarity with psychiatric diagnostic frameworks, psychotropic medication profiles, and their systemic implications (4). Conversely, psychiatrists often lacking training in ocular surface disease assessment and the interpretation of ophthalmic findings (4). This asymmetry in domain-specific expertise reinforces siloed clinical practice and constrains the effective implementation of integrated care models. Addressing these challenges will require coordinated efforts in medical education reform, interdisciplinary training, and institutional support systems.

From a health economics perspective, integrated care models may offer important long-term advantages despite these implementation challenges (79). By reducing redundant consultations—commonly described as “doctor-shopping”—as well as minimizing unnecessary ocular interventions and inefficiencies in fragmented care delivery, such models may improve both resource allocation and clinical outcomes (80, 81). This alignment with value-based care principles suggests potential system-level benefits (82). However, these advantages remain largely inferential at present, and robust cost-effectiveness analyses, including prospective and real-world studies, are required to substantiate the economic impact of integrated management strategies.

### 6.2 Future research agenda

Advancing the integrated management paradigm requires a coordinated research agenda addressing multiple knowledge gaps. Regarding underlying mechanisms, further investigation of the eye-brain axis is needed to elucidate specific molecular pathways linking ocular surface pathology with mood dysregulation (32). Identifying shared therapeutic targets—such as neuroinflammatory mediators, neurotrophic factors, or common genetic vulnerabilities—could facilitate development of interventions benefiting both conditions simultaneously (83). Regarding clinical evidence, well-designed randomized controlled trials are urgently needed to compare the integrated pathways and temporal strategies proposed here against conventional, siloed treatment approaches (1). Such trials should evaluate not only symptom reduction but also functional outcomes, quality of life, treatment adherence, and cost-effectiveness. Regarding technological innovation, developing artificial intelligence-based clinical decision support systems holds promise for guiding individualized treatment sequencing (84). By integrating longitudinal symptom data from digital monitoring tools with established phenotypic classifications, such systems could provide clinicians with real-time, personalized recommendations for temporal optimization (85). These research directions, if systematically pursued, could transform the current therapeutic landscape and establish evidence-based foundations for integrated mind-eye care.

## 7 Summary

The bidirectional comorbidity between DED and depressive disorder represents a clinical reality rather than a coincidental occurrence. Throughout this Perspective, we have synthesized evidence demonstrating that these conditions share fundamental biological pathways and psychological mechanisms, creating self-perpetuating cycles that complicate management and worsen patient outcomes. The core contribution of this article is proposing a novel integrated management paradigm that builds on mechanistic understanding, employs stratification-based phenotyping, and prioritizes temporal sequencing as a key determinant of therapeutic success. This framework challenges traditional siloed approaches by emphasizing that when interventions are delivered may be as important as which interventions are selected. We conclude by calling for a fundamental shift in medical education, clinical practice, and research design—moving from single-organ models toward integrated mind–body perspectives that recognize the inseparable nature of ocular and emotional health. Such transformation requires curricular reforms that expose trainees to interdisciplinary knowledge, practice innovations enabling genuine multidisciplinary collaboration, and research agendas evaluating outcomes through a holistic lens. Ultimately, this paradigm shift serves the overarching goal of patient-centered care that addresses the whole person rather than disconnected organ systems or diagnostic categories.

## Data Availability

The original contributions presented in the study are included in the article/supplementary material, further inquiries can be directed to the corresponding author.
